# Mapping inequalities in the health of older adults around the world: Heterogeneities in cognitive and physical functioning

**DOI:** 10.1553/p-mcm9-5b3b

**Published:** 2025-12-17

**Authors:** Thomas Arnhold, Viktoria Szenkurök, Daniela Weber

**Affiliations:** 1 POPJUS Program, International Institute for Applied Systems Analysis (IIASA), Wittgenstein Centre for Demography and Global Human Capital (IIASA, ÖAW/VID, University of Vienna). Laxenburg, Austria.; 2 Health Economics and Policy Division, Vienna University of Economics and Business (WU). Vienna, Austria.; 3 Institute for Environmental Studies, Faculty of Science, Vrije Universiteit Amsterdam (VU). Amsterdam, Netherlands.

**Keywords:** Gini index, verbal fluency, episodic memory, handgrip strength, gender, ageing

## Abstract

Amid global population ageing and evidence that health disparities in later life often stem from accumulated disadvantage, it is essential to assess health inequalities in older adults in an internationally comparable and comprehensive way. Addressing the shortcomings of analysing average health levels in a population while preserving the comparability of countries and subpopulations, we estimate Gini coefficients to examine inequalities in physical and cognitive functioning of older adults in 41 countries, stratified by gender and five-year age group. Utilising data from 11 nationally representative surveys on the health and ageing of older adults, we find substantial heterogeneities in physical and cognitive functioning inequalities across countries for both women and men. Notably, countries with higher median scores in cognitive functioning tend to exhibit significantly less pronounced inequalities. Furthermore, our results reveal a steep positive age gradient in both dimensions of cognitive functioning. Taken together, our descriptive results provide a valuable foundation for researchers and policymakers aiming to identify targeted interventions and policy measures to address health disparities.

## 1 Introduction

In the context of rapid population ageing and evidence underlining that health disparities in older age often reflect accumulated disadvantage (United Nations Department of Economic and Social Affairs, 2015, 2018), it is crucial to gain a more comprehensive understanding of existing health inequalities among older adults and how they compare across countries. Moreover, reducing inequalities is of global interest, as agreed on in Sustainable Development Goal (SDG) 10, while SDG 3 calls for ensuring healthy lives and well-being for all people at all ages (United Nations, 2016).

In this study, we use the Gini coefficient, a well-established measure in economics and related disciplines for assessing income inequalities, to evaluate inequalities in cognitive and physical functioning in 41 countries. Our analysis further stratifies these inequalities by gender and five-year age group.

The Gini index is particularly well-suited for this purpose, offering a clearly interpretable measure of total inequality solely representing the overall statistical dispersion of a variable. As such, it allows for comparisons of health disparities across subpopulations, including by age and gender, without being limited to one determinant of inequality (Wagstaff et al., 1991). Furthermore, its unit independence allows for direct comparisons of health inequalities across countries or regions, regardless of population size or wealth (Kondo et al., 2012). Because the Gini index does not rely on a reference variable, such as socio-economic status (SES), it simplifies comparisons across countries where SES or other resource measures may vary significantly or are difficult to harmonise. Moreover, the Gini index assesses inequalities in cognitive and physical functioning without assuming a specific relationship between health and other resources.

Understanding dispersions in both domains is pivotal, as maintaining high physical functioning and high cognitive functioning are two key determinants of living a healthy and independent life in older age (Rowe and Kahn, 1997). Physical functioning is defined as an individual’s ability to perform physical activities of daily living (Painter et al., 1999). Its ageing-related decline is driven by loss of muscle mass and strength, leading to difficulties in performing activities of daily living, such as self-care and homemaking, and instrumental activities of daily living, such as shopping or using transportation (Painter et al., 1999). Consequently, high physical functioning is often a prerequisite for participation in social, vocational or recreational activities (Liu, 2017; Painter et al., 1999), and is a determinant of quality of life and well-being (Gobbens and van Assen, 2014; Sentandreu-Mañó et al., 2022). Moreover, it is a predictor of all-cause mortality (Andrasfay, 2020; Cooper et al., 2010).

Cognitive functioning, on the other hand, encompasses the performance of the mental processes of perception, learning, memory, understanding, awareness, reasoning, judgment, intuition and language (American Psychological Association, 2018). Ageing, even in the absence of chronic diseases, triggers neurodegeneration, leading to decreasing cognitive functioning, predominantly affecting the basic functions *memory* and *reasoning* (Glisky, 2007; Salthouse, 2019). Similar to physical functioning, the ageing-related decline in cognitive functioning is closely linked to reduced independence and well-being in later adulthood (Salthouse, 2012).

A validated measure of physical functioning in older adults is handgrip strength (HGS) (Soysal et al., 2021), which has prognostic importance for assessing dependency in activities of daily living (Rantanen et al., 2002; Taekema et al., 2010) and mortality risk (Eekhoff et al., 2019). HGS tests are commonly used in ageing studies around the world due to their simplicity and low costs of measurement (Phillips et al., 2023). Two cognitive functioning tests widely employed in ageing studies are the immediate recall and verbal fluency tests, which measure crystallised and fluid abilities, respectively (Baltes and Lindenberger, 1997; Jaeggi et al., 2008; Nisbett et al., 2012; Shih et al., 2012).

Previous studies using common measures for cognitive and physical functioning found pronounced disparities in both domains within countries. These disparities were frequently linked to socio-economic status (SES), as indicated by factors such as income, education, parents’ education (Biritwum et al., 2016; Dodds et al., 2013; Lyu and Burr, 2016), gender (Herlitz et al., 2017; Zhong et al., 2017) or region, such as urban-rural differences (Lawrence et al., 2023; Saenz et al., 2018).

Despite these studies pointing to substantial disparities, prior research – not exclusively focusing on older adults – mainly concentrated on describing and comparing the average health levels of different (sub)populations, rather than examining the dispersion within populations (OECD/European Commission, 2024). Furthermore, prior work often focused on one health dimension, such as cognitive functioning (Lyu and Burr, 2016), physical functioning (Dodds et al., 2013) or body height (Pradhan et al., 2003). Studies investigating inequalities often focus on social inequality in health instead of total inequality (Schlotheuber and Hosseinpoor, 2022). Research using measures of total inequality in health that are comparable across countries remains scarce, and mainly focuses on cognitive functioning inequalities, rather than on physical functioning (Olivera et al., 2018; Tranvåg et al., 2013).

There is some evidence supporting the ‘expansion of morbidity’ theory, which suggests that the growing global life expectancy increases the average number of years spent in poor health, amplifying care needs and placing increasing pressure on health and social care systems (Gruenberg, 1977; Salomon et al., 2012). While health inequalities often widen with advancing age due to cumulative disadvantages over the life course (Arcaya et al., 2015; Lynch and Smith, 2005; Smith, 2007), there is evidence that this trend may not be universal. For instance, van Zon et al. (2015) showed that while relative inequalities can persist, absolute health inequalities may decrease in older age. This occurs partly due to survival selection, whereby individuals with poorer health may not survive to older age, leaving behind a healthier cohort. Similarly, Grundy and Sloggett (2003) highlighted how personal and social resources can mitigate inequalities for some older adults.

Despite some evidence suggesting that absolute inequality narrows with advancing age, older adults continue to face unique challenges, such as kinlessness (Mair, 2019), social frailty (Grundy and Sloggett, 2003; Yamada and Arai, 2018, 2023), limited access to health and social care and unmet medical needs (Kröger, 2022; Szenkurök et al., 2024). These challenges disproportionately affect disadvantaged groups, highlighting the complex and multifaceted nature of health inequalities among vulnerable populations. Furthermore, they underscore the importance of quantifying inequalities affecting older adults across countries and diverse institutional contexts.

This paper expands the literature by providing a descriptive overview of the dispersion in cognitive and physical functioning by age and gender, as well as across 41 countries. To map health inequalities among older adults, we utilise data from 11 nationally representative surveys on the health and ageing of older adults. By employing a comparable index to assess the functioning distribution of older adults, we offer a new perspective on (i) cross-national, (ii) gender- and (iii) age-related disparities in cognitive and physical functioning, thus facilitating a deeper understanding of the extent and the structure of health inequalities. As a robustness check, the results are compared to the P80/P20 ratio, another indicator of dispersion.

## 2 Data and methods

### 2.1 Data

We use data from nationally representative datasets that assess the health, social and economic conditions of non-institutionalised older adults, with the majority being sister studies of the Health and Retirement Study (HRS) (National Institute on Aging, 2007). Moreover, the main contents of the surveys are harmonised to enable cross-country comparisons (Smith, 2021). All the considered surveys include comparable performance tests on cognitive or physical functioning (Phillips et al., 2023; Shih et al., 2012), which are recognised indicators for older adults’ ability to live an independent life (Matsui et al., 2014; Salthouse, 2012). In this study, we consider the most recent waves of the following 11 surveys (presented alphabetically), which represent 41 countries across the world.

Table 1 provides a compact overview of all the surveys, countries, wave years and functioning measures (i.e., HGS, immediate recall, and verbal fluency) included in the study. We include in our sample all respondents aged between 50 and 84 who performed at least one of the three functioning tests and provided information on their age and gender, stating that they were either female or male. For data availability reasons, we have chosen 84 years as the upper bound.

Before detailing the variables used for cognitive and physical functioning, we provide in the following an overview of each of the 11 surveys, particularly focusing on their data collection design.

The Chile Cognitive Aging Study (Chile-Cog) commenced its longitudinal study in 2019. Its main objective is to measure the prevalence of dementia and cognitive ability among the Chilean population aged 60 and older. Chile-Cog follows the Harmonized Cognitive Assessment Protocol (HCAP), which was first utilised in the HRS. We use data from the 2019 Chile-Cog (Ministry of Labor and Social Welfare, 2019).

The English Longitudinal Study of Ageing (ELSA) is designed to represent the population of non-institutionalised individuals aged 50 and older living in England. The first wave was conducted in 2002, and follow-up waves were conducted biennially (Steptoe et al., 2013). Since the latest handgrip strength (HGS) assessments were performed across waves 8 and 9 (conducted in 2016 and 2018, respectively), our results are based on both waves.

The Brazilian Longitudinal Study of Aging (ELSI-Brazil), based on the HRS, is a longitudinal study of community-dwelling adults aged 50 or older living across the five great geographic regions of Brazil (Lima-Costa et al., 2022). ELSI-Brazil started in 2015 and commenced with a second release in 2019, which we use in our study.

The HRS, which was first conducted in 1992 in the United States, spans 16 finished waves (from 1992 to 2022), and is one of the earliest and most renowned longitudinal studies focusing on health, retirement and ageing (National Institute on Aging, 2007; Sonnega et al., 2014). Numerous ageing studies across the world build upon HRS and use comparable questionnaires as well as cognitive and physical functioning tests. We use data from the 15^th^ wave, collected in 2020 (Health and Retirement Study, 2023).

The Indonesia Family Life Survey (IFLS) started in 1993 and focuses on individuals aged 50 and older, including their spouses, living in Indonesia. We use data from the most recent IFLS wave 5, which was conducted between 2014 and 2015 (Strauss et al., 2016).

The Japanese Study of Aging and Retirement (JSTAR) was conducted by the Research Institute of Economy, Trade and Industry (RIETI), Hitotsubashi University, and the University of Tokyo. JSTAR encompasses longitudinal data on middle-aged and elderly Japanese individuals aged 50 and older from 2007 onwards, with the most recently released wave 3 collected in 2013 and 2014. In this article, the results are based on JSTAR wave 3.

The Longitudinal Ageing Study in India (LASI) follows older adults aged 45 and older in India (Perianayagam et al., 2022). We use the first wave, for which data were collected between 2017 and 2018, as well as between 2020 and 2021 (Chien et al., 2023).

The Mexican Health and Aging Study (MHAS) is a national longitudinal study of adults aged 50 and older in Mexico (Wong et al., 2017). The baseline survey was conducted in 2001, with follow-up interviews performed in 2003, 2012, 2015, 2018 and 2021. In this study, we use MHAS wave 3 (2012), the latest release including the HGS test, and wave 5 (2015), the latest release including the verbal fluency test (Michaels-Obregon et al., 2023).

The Study on Global AGEing and Adult Health (SAGE) collects longitudinal data on adults aged 18 and older, with an emphasis on those aged 50 and older, from six countries: China, Ghana, India, Mexico, the Russian Federation and South Africa (World Health Organization, 2006). We use the first wave, which started in 2007 and was released for all SAGE countries (Chatterji and Kowal, 2013).

The Survey of Health, Ageing and Retirement in Europe (SHARE) has collected longitudinal data every two years on individuals aged 50 and older since its launch in 2004. With its expanding coverage across Europe and Israel, SHARE now includes 28 countries (Börsch-Supan et al., 2013). In this analysis, we use data from SHARE wave 9.

The Irish Longitudinal Study on Ageing (TILDA) is a longitudinal study that collected its initial wave in 2009. It focuses non-institutionalised individuals aged 50 and older living in the Republic of Ireland (Donoghue et al., 2018). We use data from TILDA wave 1.

### 2.2 Variables: Cognitive and physical functioning

The cognitive functioning tests conducted within the ageing studies included in this study cover the two dimensions of episodic memory and verbal fluency (Shih et al., 2012). Episodic memory is measured by an immediate recall test in which the interviewer reads out a list of 10 words and asks the respondent to immediately recall as many words as possible within one minute without rereading the words. In the verbal fluency test, the interviewer asks the respondent to name as many animals as possible within 60 seconds. For comparability reasons, we top-coded all scores of respondents who named more than 20 animals to 20, given that this is the lowest upper limit of the utilised studies (in ELSI-Brazil).

As a measure of *physical functioning*, we use the performance in a HGS test. HGS in kilograms is measured using a dynamometer, either once with the dominant hand, or more often with the weaker hand as well, mainly in a standing position (Phillips et al., 2023). For comparability reasons, we use the maximum score of the individual HGS measurements.

Figures S.1–3 show the distributions of the HGS, immediate recall and verbal fluency test scores for each country.

### 2.3 Methods

In this study, we borrow an established approach from the discipline of economics to quantify health inequality. The Gini coefficient is an indicator of statistical dispersion intended to represent the inequality within a population or subpopulation (Wagstaff et al., 1991). It ranges from zero to one, with a higher Gini coefficient indicating higher inequality. We aim to measure inequality in health by the degree of inequality in the distribution of HGS, episodic memory and verbal fluency across subpopulations. To obtain inequality estimates for different subpopulations of the investigated countries, we estimate the Gini coefficient separately for the physical and cognitive functioning variables, also considering survey weights. We obtain the Gini coefficient using the estimator

(1) $$\hat{\text{Gini}}=\frac{2\sum_{i=1}^{n}w_{i}{r_{i}y}_{i}-\sum_{i=1}^{n}w_{i}y_{i}}{(\sum_{i=1}^{n}w_{i})\sum_{i=1}^{n}\left(w_{i}y_{i}\right)}-1,$$

where $y=\{y_{i},i\in U\}$ is the function distribution over the subpopulation *U* and $w_{i}$ is the survey weight, and $r_{i}$ the rank of *i* in the distribution *y*. We use the R package *convey* to obtain the Gini estimates (Djalma et al., 2023; Osier, 2009).

For each country and health measure, we estimate Gini coefficients for the subpopulation aged 50 to 84, stratified by gender and five-year age group, to gain insights into the disparities of inequalities within countries’ subpopulations. To mitigate the small sample bias of the Gini estimate, we estimate subpopulation Gini indices only for subsamples with at least 50 observations (Deltas, 2003). This exclusion criterion mostly affects the youngest five-year age group (50–54) of European countries and Israel (excluded subpopulations are indicated in Tables S.1–3).

To avoid comparability issues due to differences in survey design related to eligible age groups and small sample sizes, we additionally estimate Gini coefficients for the 60- to 74-year-old subsample, stratified by country, gender and five-year age group, to allow for better comparisons across countries.

Due to the low number of observations at the tails of the age distributions, we conduct the stratification by gender only for the *50–84*, *60–74*, *60–64*, *65–69* and *70–74* age groups.

We conduct two robustness checks. First, we address the inability of the Gini index to represent specific parts of the health distribution by comparing the Gini estimates to the P80/P20 ratio. The P80/P20 ratio, also known as the quintile share ratio, is a standard measure of inequality representing the ratio of the 80th percentile to the 20th percentile of the health distribution (European Commission, 2003; Eurostat, 2005). Compared to the Gini index, the P80/P20 ratio is less susceptible to changes in the middle of common distributions, and is less impacted by their outliers. As a second robustness check, we estimate the verbal fluency Gini indices using non-top-coded verbal fluency scores and compare them to the original estimates.

## 3 Results

Our results show substantial inequalities in cognitive and physical functioning. Inequalities in physical functioning are more pronounced than inequalities in cognitive functioning in most countries. Additionally, we observe variations in inequalities between cognitive measures, with further distinctions between immediate recall and verbal fluency (see Tables S.1–3 in the Supplementary material).

### 3.1 Country comparisons

For the population aged 50 to 84, the HGS Gini estimates indicate that inequality is lowest in Japan (0.172), followed by Finland (0.179) and Switzerland (0.180) (Table S.1), whereas inequality is highest in South Africa (0.281), Ghana (0.236) and Israel (0.234). Thus, in the HGS Gini indices, the difference between the minimum and maximum values is 0.109 Gini points (Table S.1).

For the cognitive functioning Gini estimates, we observe similar disparities between the minimum and maximum values. For the immediate recall test, the Gini estimates range from low values in England (0.148), Austria and Czechia (both at 0.149) to high values in Indonesia (0.268), Japan (0.236) and Brazil (0.235), yielding an immediate recall Gini range of 0.120 Gini points (Table S.2). Interestingly, while Japan ranks among the most equal countries for HGS, it is also among the least equal countries for immediate recall. For verbal fluency, Austria and Denmark (both at 0.045) are the countries with the most equal estimated distribution, followed by Czechia (0.048) (Table S.3). The highest verbal fluency Gini estimates are found in Russia (0.325), Brazil (0.262) and China (0.214) (Table S.3). The verbal fluency Gini estimates have the largest range, amounting to 0.280 Gini points.

**Figure 1:**
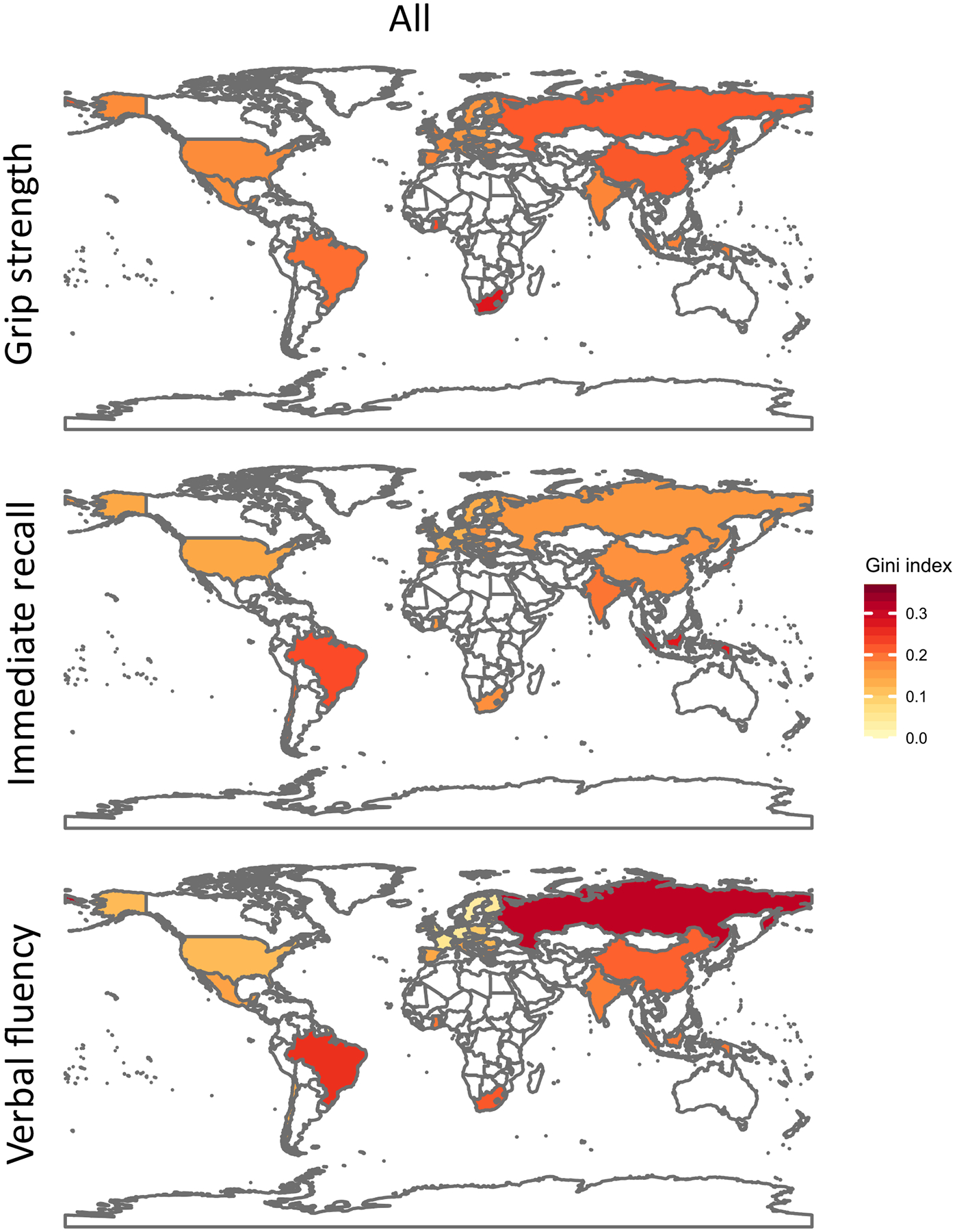
Gini estimates for handgrip strength, immediate recall, and verbal fluency of country populations aged 60 to 74.

When we limit our estimates to the populations aged 60 and 74, the country rankings of the Gini indices are largely retained (Tables S.1–3; Figure 1)

Our results also hint at substantial differences in functioning disparities across the observed countries in different continents (Figure 1). For instance, Northern European countries and the USA exhibit lower inequalities in HGS, whereas the observed African countries and China show relatively high inequalities (Table S.1; Figure 1).

Continental differences in cognitive functioning inequalities appear to be more pronounced. The observed countries in Latin America and Southeast Asia show the highest inequalities in immediate recall, pointing to continental differences in cognitive ageing processes. Similarly, verbal fluency disparities are the most pronounced in the observed Latin American and Asian countries (Tables S.2; S.3; Figure 1).

**Figure 2:**
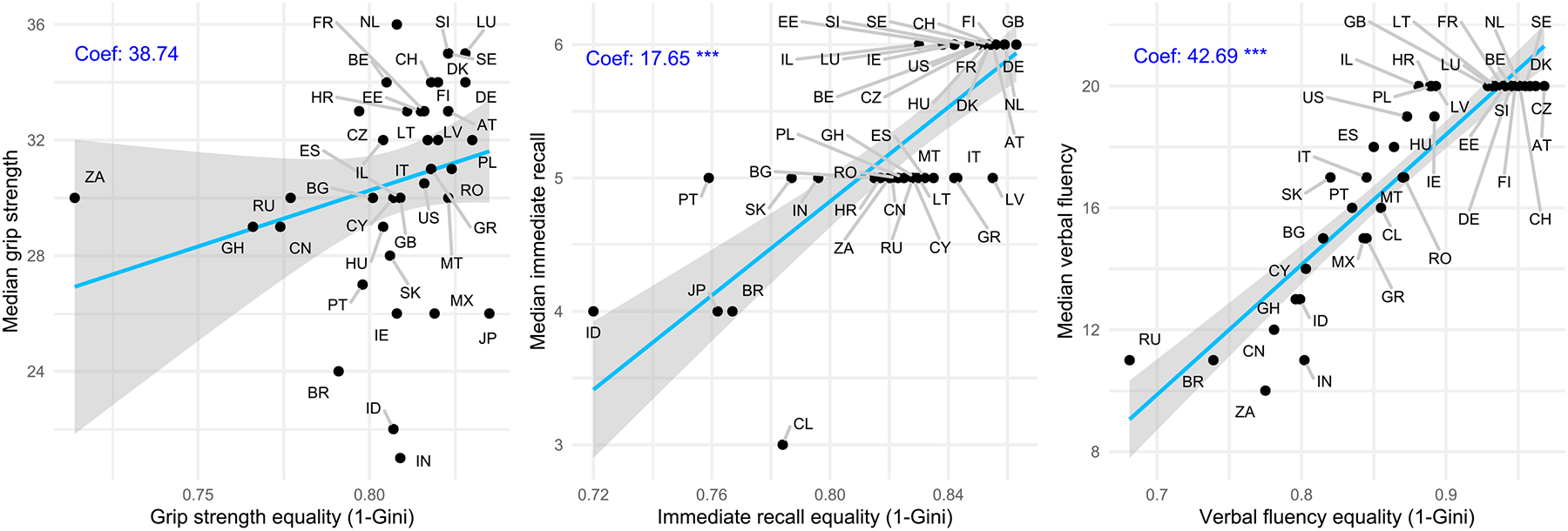
The relationship between the Gini estimates and medians of handgrip strength, immediate recall and verbal fluency for each country populations aged 60 to 74 years. Note: The x-axis represents the health equality, measured as $1-\hat{\text{Gini}}$. The y-axis represents the estimated country median. The upper left of the tables shows the coefficients and their significance levels when regressing the median on $1-\hat{\text{Gini}}$ (Confidence levels: * p<0.05; ** p<0.01; *** p<0.001). The solid line represents a regression line, with the grey area denoting the standard errors. Austria = AT, Belgium = BE, Brazil = BR, Bulgaria = BG, Chile = CL, China = CN, Croatia = HR, Cyprus = CY, Czechia = CZ, Denmark = DK, England = GB, Estonia = EE, Finland = FI, France = FR, Germany = DE, Ghana = GH, Greece = GR, Hungary = HU, India = IN, Indonesia = ID, Ireland = IE, Israel = IL, Italy = IT, Japan = JP, Latvia = LV, Lithuania = LT, Luxembourg = LU, Malta = MT, Mexico = MX, Netherlands = NL, Poland = PL, Portugal = PT, Romania = RO, Russia = RU, Slovakia = SK, Slovenia = SI, South Africa = ZA, Spain = ES, Sweden = SE, Switzerland = CH, USA = US.

Interestingly, the countries with lower cognitive and physical functioning Gini estimates show higher weighted median functioning scores (Figure 2). For the two cognitive functioning domains, we find statistically significant positive correlations between estimated equalities (1-Gini) and the weighted medians (p<0.001). For HGS, the positive correlation is not statistically significant (see Figure 2).

### 3.2 Gender differences

**Figure 3:**
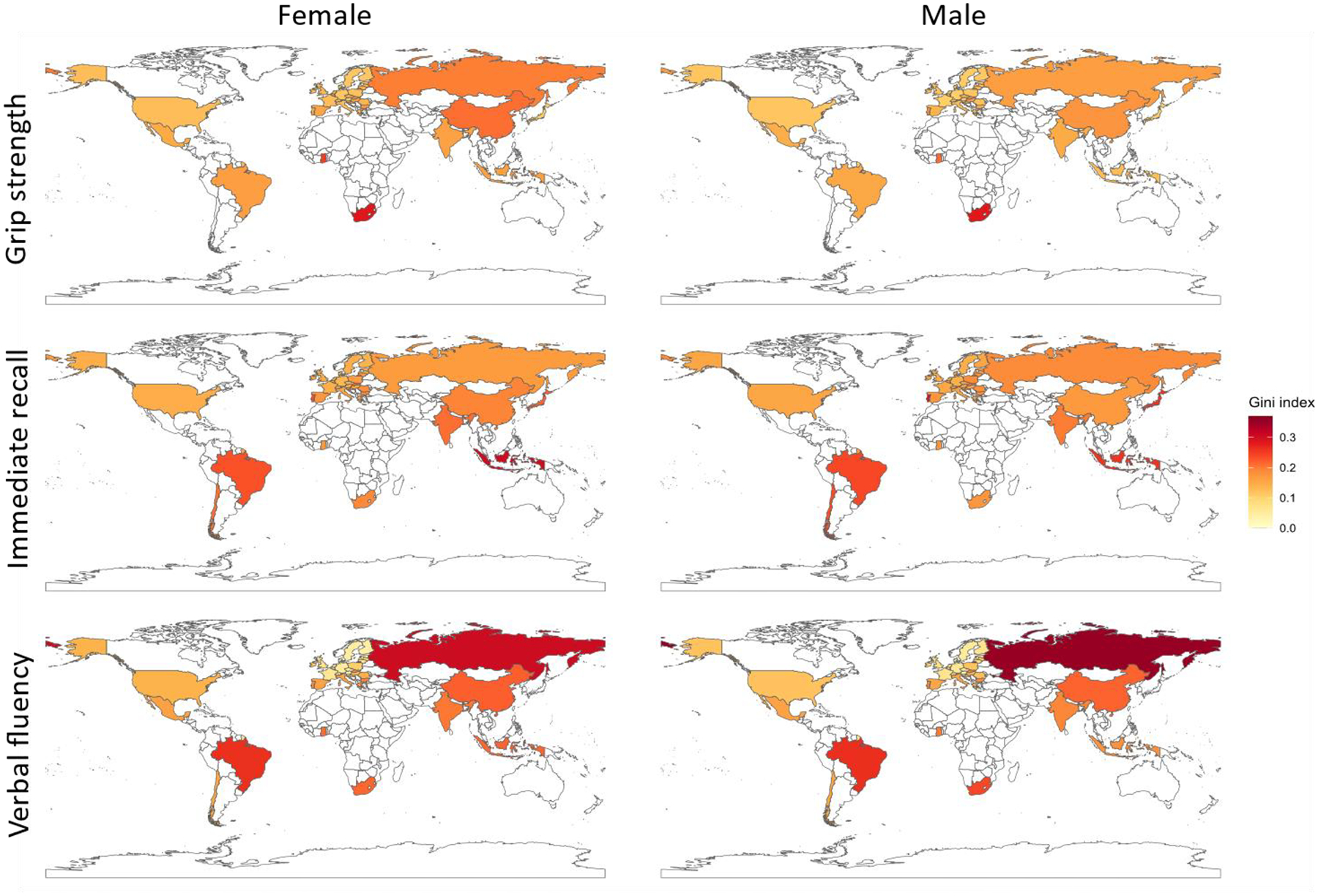
Gini estimates for handgrip strength, immediate recall, and verbal fluency of country populations aged 60 to 74 by gender.

For most countries, we estimate that women experience greater inequalities in HGS, whereas men exhibit greater inequalities in both immediate recall and verbal fluency. These observations hold true for the populations aged 50 to 84 as well as for those around retirement age (60–74 years old) (Tables S.1–3; Figure 3).

Gender differences in the HGS Gini estimates are substantial in Bulgaria, China, Ghana and Russia, where we estimate higher inequality for women than for men, and in Israel, where inequality is higher for men than for women.

For the immediate recall Gini estimates, most countries show minimal gender differences, with the notable exceptions of Japan, where men record a higher estimated immediate recall Gini than women, and Israel, where the opposite pattern is observed. Moreover, a substantially higher verbal fluency Gini estimate for women than for men is found in Israel.

### 3.3 Age group differences

The analysis of the Gini estimates by age group suggests that there is a U-shaped relationship between the HGS inequalities and age. Specifically, the median HGS Gini across countries for the *50–54* age group is higher than that for the subsequent age groups of *55–59*, *60–64* and *65–69*. Beyond age 54, we observe a positive age group gradient. From the youngest (*50–54*) to the oldest (*80–84*) age group, the percentual increase of the median HGS Gini is 9.59% (from 0.188 to 0.206) (Figure S.4).

The immediate recall and verbal fluency Gini estimates show a positive age group gradient. This gradient is most pronounced for verbal fluency, with a substantial increase in the median Gini from the *50–54* to the *80–84* age group of 111.48% (from 0.097 to 0.206), compared to a 55.14% increase for episodic memory (from 0.150 to 0.233) (Figure S.4). Thus, the estimated median health inequality within five-year age groups is higher for the most advanced age group than for the younger age groups across all countries, highlighting pronounced heterogeneities among the oldest-old (Figure S.4 in the Supplementary material).

Table 2 compares age group differences in the Gini estimates for the two genders separately, focusing on the *60–64*, *65–69* and *70–74* age groups. For women, we observe a consistent positive age-related gradient in the country median Gini estimates for both physical and cognitive functioning. For men, the *65–69* age group records the lowest estimates, followed by the *60–64* age group, with the *70–74* age group exhibiting the highest values across all outcomes.

Within the age groups, the median HGS Gini estimates are consistently higher for women than for men. In contrast, the median immediate recall Gini estimates are higher for men than for women. For verbal fluency, women exhibit lower median Gini estimates in the *60–64* age group but record higher values in the older *65–69* and *70–74* age groups compared to their male counterparts.

### 3.4 Robustness analyses

Both robustness analyses — substituting the Gini coefficient with the P80/P20 ratio and removing top-coding from the verbal fluency test score — point to the stability of our results.

When comparing the Gini with the P80/P20 ratio at the country level, we observe a highly significant positive correlation (p<0.001) and no meaningful shifts in the country rankings between the measures (See Figure S.5 in the Supplementary material).

Similarly, using the non-top-coded version of the verbal fluency score does not substantially alter the ranking of most countries, with the two measures showing a highly significant positive correlation (p<0.001). Although the non-top-coded version of the verbal fluency score has little impact on the overall order of countries, it affects the magnitude of the Gini coefficient, particularly for countries with high median scores (see Table S.4 and Figure S.6 in the Supplementary material).

## 4 Discussion

Using the most recent data from 11 nationally representative surveys on the health and ageing of older adults in 41 countries, this study expands the literature on heterogeneities in cognitive and physical functioning. Distinguishing between (i) cross-national, (ii) gender- and (iii) age-related disparities in cognitive and physical functioning, we estimate not only the levels, as was done in the previous literature (e.g., Lyu and Burr, 2016), but also the distribution of older adults’ health using the Gini index.

Our results show that, across the indicators used to describe cognitive and physical functioning, countries with higher average health levels among older adults tend to exhibit a more equal distribution in these measures. While this relationship is statistically significant for the two cognitive functioning domains (p<0.001), it is not statistically significant for HGS.

These positive relationships are likely influenced by confounding factors. For instance, European countries, which tend to exhibit higher scores, are largely characterised by higher levels of social protection and universal healthcare coverage, leading to more equitable access to health services (International Labour Organization, 2024). These factors contribute to healthier lifestyles and better health outcomes for many people, potentially resulting in a more equal distribution of health.

At the country level, we observe considerable differences in the magnitude of inequality between cognitive and physical functioning measures, with inequality being greater for HGS than for episodic memory (i.e., immediate recall) or verbal fluency. This observation is partly explainable by the consistently large variation in HGS due to gender, likely stemming from biological differences in muscle mass and differences in physical activity between the sexes (Dhara et al., 2011; Huebner et al., 2022).

Besides gender differences in levels, we observe that the HGS Gini tends to be higher for women than for men, suggesting greater variation in physical functioning among women. This may reflect greater differences in lifestyle, particularly in terms of lifetime engagement in physically demanding activities (Dodds et al., 2013).

In contrast, we observe greater inequality in cognitive functioning among men, particularly in immediate recall and verbal fluency. Research suggests that this disparity may stem from greater male variability in educational attainment, leading to differences in intellectual experiences throughout working ages that have a protective effect on cognitive functioning later in life (O’Dea et al., 2018; Okamoto et al., 2021).

Furthermore, within countries, our results reveal substantial inequalities by age group, indicating that inequalities are greater among the most advanced age groups. We find a U-shaped relationship between age and inequality in physical functioning, with inequalities initially decreasing from the *50–54* to the *55–59* age group, and then increasing again. We also find a strongly positive age gradient for cognitive functioning, which is most pronounced for verbal fluency. Notably, the initial decrease in the Gini estimate from the *50–54* to the *55–59* age group may stem from selection bias due to the exclusion of 10 European countries and Israel from the *50–54* subsample due to low sample sizes, given that these countries often exhibit lower HGS Gini values for the other subgroups.

Indeed, prior research has shown that adverse environmental conditions during infancy and early childhood, along with subsequent life events, contribute to chronic disease risk in adulthood, potentially exacerbating health inequalities with advancing age (Lynch and Smith, 2005; Smith, 2007). In particular, the literature highlights that disadvantaged older adults face distinct challenges, such as social frailty, which stems from a lack of social capital, including family relationships (Grundy and Sloggett, 2003; Yamada and Arai, 2018, 2023), and is associated with lower levels of both cognitive and physical functioning (Lee et al., 2024; Ma et al., 2018).

Overall, our results align with the existing literature, demonstrating that disparities in health among older adults vary by age group and gender (Dhara et al., 2011; Huebner et al., 2022; Zon et al., 2015), as well as across countries (Barber et al., 2017; Beckfield and Olafsdottir, 2013; MacKinnon et al., 2023; Steinbeis et al., 2019).

The particular vulnerability of the oldest segment of the population to both cognitive and physical functioning decline, coupled with demographic shifts and associated changes in family and household structures (Grundy and Sloggett, 2003), underscores the need to describe inequalities within this group and provide a comprehensive picture of the magnitude and extent of these inequalities. By providing a globally comparable overview of total health inequalities, we addressed this gap, laying the groundwork for future research on the causal mechanisms behind health disparities in older adults within specific national or sub-national contexts, as well as for policymakers aiming to mitigate emerging inequalities.

### 4.1. Limitations

Using the most recent available releases of a wide array of surveys, we mapped inequalities in various health dimensions critical for maintaining an independent life across a uniquely broad geographical scope. While the surveys were conducted in different time periods, which may place some constraints on their comparability, they have been harmonised following the HRS framework, specifically to facilitate cross-country comparisons (Smith, 2021). However, differences in the HGS assessment could introduce biases. Most surveys used the same dynamometer, but a few used different brands and allowed measurements in a seated position (Phillips et al., 2023).

Furthermore, variations in survey designs could limit the comparability of the results. For instance, the Chile-Cog survey only includes older adults aged 60 and older and several countries in SHARE have a low number of observations for the youngest age group (*50–54*), which may lead to bias in the dispersion of functioning scores. We addressed this issue by conducting country comparisons for the broader 60–75 age group, as the age distributions across countries are similar for this age group. Furthermore, the subsample of 60–75-year-olds reduces sample selection due to the survival of healthier older adults. Another important limitation is that while our sample includes 41 countries, it still only represents a subset of the world’s nations.

Additionally, while the Gini index provides notable advantages as a measure of inequality for the purposes of our study, it also has limitations. The Gini index offers advantages over other indicators, such as the Concentration Index that was commonly used in previous studies focusing on health inequalities (Siegel and Allanson, 2016; Wagstaff et al., 1991), as it measures inequality purely within the health outcomes of interest (i.e., cognitive or physical functioning), making it well-suited for comparisons across countries and survey designs (Steinbeis et al., 2019).

It is, however, crucial to emphasise a major limitation of the Gini index: i.e., its limited ability to represent specific parts of the health distribution. We addressed this issue by including the P80/P20 ratio as a robustness check, specifically focusing on disparities between two specific parts of the distribution, which showed similar results.

Another consequence of this limitation is that as a measure of dispersion, the Gini cannot distinguish whether variation in the distribution occurs on its upper or its lower end. Thus, the Gini index does not fulfil the property of transfer sensitivity, meaning that it is not necessarily more sensitive to health improvements at the lower end of the distribution than to health deterioration at the upper end (Foster et al., 2013).

## 5 Conclusions

Due to population ageing, health inequalities among older adults represent a growing concern in many countries. However, studies that use easy-to-read and objective measures to adequately reflect health inequalities among the population of older adults while allowing for cross-country comparisons are rare. Using the Gini index to assess inequalities in cognitive and physical functioning among older adults within and across 41 countries worldwide, this study complements earlier research emphasising geographical heterogeneities in health.

The main findings of our study are threefold. First, country comparisons reveal greater cross-country variation in estimated inequalities in cognitive functioning than in physical functioning, with a greater spread for verbal fluency than for episodic memory. Second, we find considerable gender differences in functioning inequalities, hinting at higher inequalities in physical functioning but lower inequalities in cognitive functioning among men compared to women. Third, our results show that older age groups tend to exhibit higher inequalities in physical functioning and, most notably, in cognitive functioning, which suggests that inequalities accumulate with advancing age.

While health inequalities may result from various individual, societal and institutional factors, such as cultural differences in health behaviour or access to health and social care, which policymakers may consider within their country-specific contexts, this article provides the groundwork for describing total inequalities among older adults. By investigating health distributions across populations and subpopulations, particularly by age and gender, this study underscores the value of internationally comparable objective health measures for cross-national and sub-national comparisons.

Researchers should leverage these opportunities in their future work to deepen the understanding of global health inequalities. Additionally, further investigations should employ inequality measures that focus on disparities at the lower end of the health spectrum, where targeted interventions and redistribution are most urgently needed.

## Supplementary Material

Supplementary material

Available online at https://doi.org/10.1553/p-mcm9-5b3b

Supplementary file 1. Tables S.1–S.4, Figures S.1–S.6

## Figures and Tables

**Table 1:** Overview of all surveys used in this study (ordered in alphabetically) providing information on country, wave with survey year and descriptive statistics (mean, standard deviation, median, and sample size N) for handgrip strength, episodic memory, and verbal fluency.

Study	Country	Wave (Year)	Handgrip strength Mean (SD) [Median] N	Episodic Memory Mean (SD) [Median] N	Verbal Fluency Mean (SD) [Median] N
Chile-Cog	Chile	Health and Cognition among Older Adults 2019 (2017, 2019)	✗	3.31 (1.37) [3] N=1,793	14.62 (4.22) [15] N=1,848
ELSA	England	Wave 8 (2016/17)	30.26 (10.73) [28.0] N=3,142		
		Wave 9 (2018/19)	30.63 (10.75) [29.0] N=2,847	6.07 (1.73) [6] N=6,474	18.21 (3.54) [20] N=6,504
ELSI-Brazil	Brazil	Wave 2 (2019)	24.32 (9.47) [23.0] N=7,619	3.95 (1.77) [4] N=8,253	10.61 (4.86) [10] N=7,977
HRS	United States of America	Wave 15 (2020)	31.09 (10.37) [29.5] N=5,952	5.62 (1.66) [6] N=13,490	15.57 (4.60) [17] N=6,520
IFLS	Indonesia	Wave 5 (2014/15)	25.53 (8.75) [24.0] N=6,359	3.83 (1.79) [4] N=6,597	13.43 (4.44) [13] N=6,591
JSTAR	Japan	JSTAR Survey 2013 (2013/14)	27.05 (8.06) [25.0] N=3,593	4.18 (1.80) [4] N=3,171	✗
LASI	India	Wave 1 (Version A.3) (2017/18, 2020/21)	23.14 (8.07) [22.0] N=44,917	4.97 (1.83) [5] N=51,186	11.30 (4.10) [11] N=51,183
MHAS	Mexico	Wave 3 (2012)	26.15 (8.97) [26.0] N=1,875	✗	
		Wave 5 (2018)		✗	15.12 (4.19) [16] N=14,026
SAGE	China, Ghana, Russia, South Africa	Wave 1 (2007–2010)	31.83 (15.12) [30.0] N=17,338	5.11 (1.69) [5] N=19,373	11.98 (4.86) [12] N=19,340
SHARE	Austria, Belgium, Bulgaria, Croatia, Cyprus, Czechia, Denmark, Estonia, Finland, France, Germany, Greece, Hungary, Israel, Italy, Latvia, Lithuania, Luxembourg, Malta, Netherlands, Poland, Portugal, Romania, Slovakia, Slovenia, Spain, Sweden, Switzerland	Wave 9 (2021/22)	32.84 (11.19) [31.0] N=58,059	5.39 (1.73) [5] N=62,275	17.41 (3.93) [20] N=62,204
TILDA	Republic of Ireland	Wave 1 (2009–2011)	28.12 (10.04) [26.0] N=5,758	5.69 (1.68) [6] N=6,297	17.34 (3.69) [20] N=8,122

Note: The ✗ indicates variables that were not available or standardizable in the study. Verbal fluency scores are top-coded at 20.

**Table 2:** Median Gini estimates for HGS, immediate recall, and verbal fluency across countries, stratified by gender and age group (for the age groups 60–64, 65–69, and 70–74).

Age group	Grip strength		Immediate recall		Verbal fluency	
	*Female*	*Male*	*Female*	*Male*	*Female*	*Male*
*60–64*	0.131	0.122	0.153	0.167	0.103	0.120
*65–69*	0.135	0.118	0.164	0.166	0.130	0.116
*70–74*	0.137	0.130	0.176	0.185	0.152	0.139

Note: The restriction to the three age groups *60–64*, *65–69*, and *70–74* is due to sample size constraints.
