# Supplementary material for: Mapping inequalities in the health of older adults around the world: Heterogeneities in cognitive and physical functioning
Source: Vienna Yearb Popul Res. Author manuscript; Available in PMC 2026 May 13. (PMC13166111; doi:10.1553/p-mcm9-5b3b)
Supplement: Supplementary material [file NIHMS2171319-supplement-Supplementary_material.docx]

Supplementary Material

Supplement to: Arnhold, T., Szenkurök, V., and Weber, D. (2025). Mapping inequalities in the health of older adults around the world: Heterogeneities in cognitive and physical functioning. *Vienna Yearbook of Population Research*, 23. https://doi.org/10.1553/p-mcm9-5b3b

Table S.1: Handgrip strength Gini estimates of the 50–84-year-olds, women 50-84 years old and men 50-84 years old, 60–74-year-olds, women 60-74 years old and men 60-74 years old, as well as by 5-year age groups between 50 and 84 for each country.

| **Country** | **50-84** | **50-84 Female** | **50-84 Male** | **60-74** | **60-74 Female** | **60-74 Male** | **50-54** | **55-59** | **60-64** | **65-69** | **70-74** | **75-79** | **80-84** |
| --- | --- | --- | --- | --- | --- | --- | --- | --- | --- | --- | --- | --- | --- |
| Austria | 0.190 | 0.134 | 0.122 | 0.177 | 0.114 | 0.110 | 0.184 | 0.179 | 0.177 | 0.175 | 0.175 | 0.185 | 0.200 |
| Belgium | 0.208 | 0.155 | 0.143 | 0.195 | 0.139 | 0.125 | 0.209 | 0.212 | 0.195 | 0.195 | 0.191 | 0.198 | 0.201 |
| Brazil | 0.216 | 0.171 | 0.157 | 0.209 | 0.163 | 0.149 | 0.201 | 0.214 | 0.202 | 0.208 | 0.219 | 0.220 | 0.235 |
| Bulgaria | 0.210 | 0.184 | 0.149 | 0.199 | 0.174 | 0.152 | NA | 0.206 | 0.199 | 0.189 | 0.226 | 0.227 | 0.236 |
| Chile | NA | NA | NA | NA | NA | NA | NA | NA | NA | NA | NA | NA | NA |
| China | 0.229 | 0.215 | 0.177 | 0.226 | 0.208 | 0.174 | 0.216 | 0.225 | 0.220 | 0.226 | 0.237 | 0.248 | 0.274 |
| Croatia | 0.202 | 0.151 | 0.127 | 0.189 | 0.137 | 0.117 | 0.182 | 0.181 | 0.185 | 0.181 | 0.192 | 0.206 | 0.222 |
| Cyprus | 0.197 | 0.153 | 0.137 | 0.193 | 0.142 | 0.123 | NA | 0.179 | 0.197 | 0.192 | 0.198 | 0.209 | 0.194 |
| Czechia | 0.195 | 0.140 | 0.146 | 0.203 | 0.145 | 0.144 | 0.200 | 0.230 | 0.230 | 0.184 | 0.192 | 0.192 | 0.192 |
| Denmark | 0.188 | 0.127 | 0.118 | 0.180 | 0.111 | 0.108 | 0.173 | 0.176 | 0.176 | 0.186 | 0.174 | 0.185 | 0.189 |
| England | 0.199 | 0.142 | 0.131 | 0.191 | 0.129 | 0.119 | 0.188 | 0.188 | 0.191 | 0.190 | 0.190 | 0.200 | 0.198 |
| Estonia | 0.198 | 0.141 | 0.124 | 0.185 | 0.129 | 0.118 | 0.188 | 0.182 | 0.179 | 0.181 | 0.191 | 0.179 | 0.204 |
| Finland | 0.179 | 0.118 | 0.116 | 0.177 | 0.111 | 0.109 | NA | 0.165 | 0.184 | 0.173 | 0.172 | 0.172 | 0.186 |
| France | 0.196 | 0.135 | 0.118 | 0.184 | 0.124 | 0.105 | 0.196 | 0.185 | 0.185 | 0.176 | 0.190 | 0.199 | 0.211 |
| Germany | 0.182 | 0.124 | 0.120 | 0.172 | 0.112 | 0.105 | 0.176 | 0.171 | 0.170 | 0.173 | 0.170 | 0.173 | 0.187 |
| Ghana | 0.236 | 0.244 | 0.211 | 0.234 | 0.244 | 0.211 | 0.204 | 0.234 | 0.227 | 0.217 | 0.260 | 0.247 | **0.321** |
| Greece | 0.193 | 0.149 | 0.149 | 0.182 | 0.133 | 0.135 | NA | 0.186 | 0.184 | 0.176 | 0.184 | 0.188 | 0.216 |
| Hungary | 0.200 | 0.155 | 0.172 | 0.196 | 0.156 | 0.165 | 0.190 | 0.190 | 0.180 | 0.193 | 0.208 | 0.209 | 0.210 |
| India | 0.196 | 0.160 | 0.149 | 0.191 | 0.158 | 0.146 | 0.177 | 0.182 | 0.182 | 0.188 | 0.199 | 0.211 | 0.219 |
| Indonesia | 0.194 | 0.154 | 0.133 | 0.193 | 0.161 | 0.125 | 0.185 | 0.180 | 0.181 | 0.188 | 0.214 | 0.207 | 0.229 |
| Ireland | 0.205 | 0.140 | 0.134 | 0.192 | 0.125 | 0.121 | 0.194 | 0.194 | 0.191 | 0.187 | 0.198 | 0.191 | 0.214 |
| Israel | 0.234 | 0.145 | 0.195 | 0.196 | 0.141 | 0.120 | NA | NA | NA | 0.206 | 0.194 | 0.226 | 0.276 |
| Italy | 0.184 | 0.133 | 0.131 | 0.182 | 0.126 | 0.130 | NA | 0.177 | 0.179 | 0.178 | 0.185 | 0.188 | 0.187 |
| Japan | **0.172** | **0.113** | **0.115** | **0.165** | **0.103** | 0.103 | 0.191 | 0.167 | 0.163 | **0.161** | **0.166** | **0.164** | 0.190 |
| Latvia | 0.186 | 0.144 | **0.115** | 0.18 | **0.137** | 0.117 | 0.168 | 0.166 | 0.163 | 0.199 | 0.179 | 0.192 | 0.202 |
| Lithuania | 0.188 | 0.145 | 0.134 | 0.183 | 0.137 | 0.132 | NA | 0.170 | 0.179 | 0.184 | 0.191 | 0.186 | 0.213 |
| Luxembourg | 0.190 | 0.144 | 0.119 | 0.172 | 0.122 | **0.100** | NA | NA | 0.172 | 0.179 | 0.180 | 0.193 | NA |
| Malta | 0.198 | 0.141 | 0.127 | 0.177 | 0.124 | 0.106 | NA | 0.198 | 0.186 | 0.178 | 0.179 | 0.185 | 0.222 |
| Mexico | 0.185 | 0.152 | 0.149 | 0.181 | 0.147 | 0.142 | 0.189 | 0.176 | 0.178 | 0.199 | 0.177 | 0.211 | 0.208 |
| Netherlands | 0.184 | 0.139 | 0.128 | 0.192 | 0.141 | 0.111 | NA | 0.206 | 0.209 | 0.173 | 0.177 | 0.194 | **0.179** |
| Poland | 0.182 | 0.131 | 0.119 | 0.170 | 0.12 | 0.111 | 0.177 | 0.169 | **0.160** | 0.172 | 0.175 | 0.193 | 0.204 |
| Portugal | 0.188 | 0.157 | 0.132 | 0.202 | 0.137 | 0.173 | 0.235 | 0.176 | **0.247** | 0.179 | 0.248 | 0.201 | 0.211 |
| Romania | 0.182 | 0.144 | 0.123 | 0.176 | 0.133 | 0.117 | **0.153** | **0.160** | 0.172 | 0.177 | 0.186 | 0.208 | 0.219 |
| Russia | 0.230 | 0.195 | 0.166 | 0.223 | 0.195 | 0.163 | 0.188 | 0.221 | 0.221 | 0.222 | 0.225 | 0.255 | 0.256 |
| Slovakia | 0.201 | 0.180 | 0.179 | 0.194 | 0.168 | 0.176 | NA | 0.186 | 0.188 | 0.207 | 0.192 | 0.240 | NA |
| Slovenia | 0.190 | 0.132 | 0.120 | 0.177 | 0.119 | 0.107 | 0.174 | 0.187 | 0.174 | 0.172 | 0.178 | 0.182 | 0.189 |
| South Africa | **0.281** | **0.280** | **0.268** | **0.286** | **0.284** | **0.279** | **0.270** | **0.273** | **0.273** | **0.305** | **0.289** | **0.307** | **0.321** |
| Spain | 0.199 | 0.164 | 0.136 | 0.191 | 0.151 | 0.135 | 0.195 | 0.180 | 0.184 | 0.206 | 0.201 | 0.209 | 0.202 |
| Sweden | 0.187 | 0.131 | **0.115** | 0.177 | **0.112** | 0.107 | 0.182 | 0.180 | 0.180 | 0.176 | 0.175 | 0.180 | 0.197 |
| Switzerland | 0.180 | 0.127 | 0.117 | 0.182 | 0.117 | 0.115 | NA | 0.163 | 0.182 | 0.177 | 0.179 | 0.185 | 0.195 |
| USA | 0.187 | 0.132 | 0.123 | 0.184 | 0.124 | 0.116 | 0.179 | 0.175 | 0.179 | 0.183 | 0.186 | 0.189 | 0.191 |

Note: Minimum and maximum values in each column are in bold letters. NAs are entered for subsamples including less than 50 observations.

Table S.2: Immediate recall Gini estimates of the 50–84-year-olds, women 50-84 years old and men 50-84 years old, 60–74-year-olds, women 60-74 years old and men 60-74 years old, as well as by 5-year age groups between 50 and 84 for each country.

| **Country** | **50-84** | **50-84 Female** | **50-84 Male** | **60-74** | **60-74 Female** | **60-74 Male** | **50-54** | **55-59** | **60-64** | **65-69** | **70-74** | **75-79** | **80-84** |
| --- | --- | --- | --- | --- | --- | --- | --- | --- | --- | --- | --- | --- | --- |
| Austria | 0.149 | **0.143** | 0.156 | 0.145 | 0.139 | 0.152 | 0.138 | 0.132 | 0.135 | 0.142 | 0.166 | 0.168 | 0.192 |
| Belgium | 0.159 | 0.154 | 0.163 | 0.149 | 0.141 | 0.156 | 0.153 | 0.141 | 0.134 | 0.143 | 0.170 | 0.174 | 0.225 |
| Brazil | 0.235 | 0.233 | 0.236 | 0.233 | 0.229 | 0.237 | 0.223 | 0.218 | 0.228 | 0.23 | 0.245 | 0.253 | 0.302 |
| Bulgaria | 0.188 | 0.197 | 0.181 | 0.185 | 0.191 | 0.185 | NA | 0.191 | 0.174 | 0.187 | 0.211 | 0.189 | 0.246 |
| Chile | 0.222 | 0.212 | 0.234 | 0.216 | 0.205 | 0.230 | NA | NA | 0.206 | 0.221 | 0.232 | 0.235 | 0.292 |
| China | 0.182 | 0.190 | 0.173 | 0.179 | 0.190 | 0.168 | 0.163 | 0.167 | 0.163 | 0.184 | 0.201 | 0.233 | 0.275 |
| Croatia | 0.202 | 0.204 | 0.201 | 0.183 | 0.176 | 0.191 | 0.168 | 0.17 | 0.172 | 0.174 | 0.206 | 0.241 | 0.289 |
| Cyprus | 0.180 | 0.192 | 0.172 | 0.172 | 0.177 | 0.174 | NA | 0.143 | 0.180 | 0.162 | 0.195 | 0.250 | 0.278 |
| Czechia | 0.149 | 0.156 | **0.147** | 0.148 | 0.138 | 0.158 | 0.146 | 0.229 | 0.150 | 0.146 | 0.155 | 0.166 | **0.162** |
| Denmark | 0.154 | 0.149 | 0.156 | 0.146 | 0.141 | 0.149 | 0.135 | 0.141 | 0.137 | 0.138 | 0.166 | 0.178 | 0.188 |
| England | **0.148** | 0.146 | 0.150 | 0.141 | 0.140 | **0.141** | 0.131 | **0.119** | 0.138 | **0.132** | 0.153 | 0.187 | 0.197 |
| Estonia | 0.163 | 0.156 | 0.169 | 0.158 | 0.143 | 0.176 | 0.148 | 0.132 | 0.134 | 0.162 | 0.180 | 0.167 | 0.218 |
| Finland | 0.154 | 0.145 | 0.162 | 0.144 | 0.135 | 0.152 | NA | 0.137 | 0.157 | 0.141 | 0.140 | **0.154** | 0.233 |
| France | 0.153 | 0.149 | 0.157 | 0.146 | 0.141 | 0.149 | 0.141 | 0.141 | 0.138 | 0.145 | 0.157 | 0.171 | 0.205 |
| Germany | 0.151 | 0.148 | 0.151 | **0.137** | **0.131** | **0.141** | **0.130** | 0.141 | 0.134 | 0.138 | 0.140 | 0.165 | 0.192 |
| Ghana | 0.175 | 0.177 | 0.173 | 0.171 | 0.171 | 0.172 | 0.165 | 0.166 | 0.161 | 0.175 | 0.181 | 0.195 | 0.228 |
| Greece | 0.174 | 0.182 | 0.167 | 0.158 | 0.158 | 0.161 | 0.143 | 0.152 | 0.150 | 0.153 | 0.167 | 0.195 | 0.219 |
| Hungary | 0.153 | 0.150 | 0.159 | 0.147 | 0.148 | 0.149 | 0.145 | 0.144 | 0.147 | 0.139 | 0.164 | 0.172 | 0.204 |
| India | 0.201 | 0.206 | 0.194 | 0.204 | 0.208 | 0.198 | 0.182 | 0.185 | 0.193 | 0.205 | 0.219 | 0.238 | 0.259 |
| Indonesia | **0.268** | **0.278** | **0.256** | **0.280** | **0.299** | 0.255 | **0.242** | **0.254** | 0.264 | **0.283** | **0.300** | **0.303** | 0.333 |
| Ireland | 0.169 | 0.172 | 0.165 | 0.158 | 0.155 | 0.161 | 0.134 | 0.140 | 0.149 | 0.154 | 0.173 | 0.203 | 0.229 |
| Israel | 0.205 | 0.243 | 0.195 | 0.170 | 0.184 | 0.166 | NA | NA | NA | 0.174 | 0.181 | 0.244 | **0.348** |
| Italy | 0.171 | 0.165 | 0.180 | 0.157 | 0.157 | 0.159 | NA | 0.159 | 0.140 | 0.164 | 0.174 | 0.201 | 0.231 |
| Japan | 0.236 | 0.218 | **0.256** | 0.238 | 0.217 | 0.255 | 0.202 | 0.205 | 0.226 | 0.232 | 0.254 | 0.266 | 0.327 |
| Latvia | 0.153 | 0.159 | **0.147** | 0.145 | 0.144 | 0.149 | 0.138 | 0.140 | 0.135 | 0.139 | 0.167 | 0.176 | 0.199 |
| Lithuania | 0.182 | 0.181 | 0.180 | 0.170 | 0.164 | 0.175 | NA | 0.157 | 0.173 | 0.162 | 0.182 | 0.228 | 0.275 |
| Luxembourg | 0.170 | 0.165 | 0.191 | 0.162 | 0.148 | 0.178 | NA | NA | 0.161 | 0.156 | 0.181 | 0.230 | NA |
| Malta | 0.181 | 0.177 | 0.184 | 0.165 | 0.160 | 0.173 | NA | 0.178 | 0.155 | 0.170 | 0.184 | 0.223 | 0.286 |
| Mexico | NA | NA | NA | NA | NA | NA | NA | NA | NA | NA | NA | NA | NA |
| Netherlands | 0.155 | 0.159 | 0.163 | 0.141 | 0.139 | 0.146 | NA | 0.196 | 0.151 | 0.138 | **0.139** | 0.184 | 0.185 |
| Poland | 0.188 | 0.186 | 0.189 | 0.175 | 0.165 | 0.185 | 0.196 | 0.169 | 0.172 | 0.165 | 0.188 | 0.204 | 0.272 |
| Portugal | 0.234 | 0.234 | 0.249 | 0.241 | 0.227 | **0.297** | 0.231 | 0.195 | **0.273** | 0.202 | **0.300** | 0.297 | 0.301 |
| Romania | 0.181 | 0.180 | 0.185 | 0.177 | 0.179 | 0.178 | 0.166 | 0.147 | 0.163 | 0.168 | 0.218 | 0.221 | 0.306 |
| Russia | 0.180 | 0.173 | 0.193 | 0.172 | 0.167 | 0.185 | 0.162 | 0.148 | 0.145 | 0.179 | 0.190 | 0.226 | 0.194 |
| Slovakia | 0.214 | 0.225 | 0.203 | 0.213 | 0.219 | 0.208 | NA | 0.181 | 0.205 | 0.210 | 0.239 | 0.239 | 0.294 |
| Slovenia | 0.162 | 0.162 | 0.163 | 0.153 | 0.148 | 0.159 | 0.132 | 0.143 | 0.142 | 0.159 | 0.157 | 0.181 | 0.238 |
| South Africa | 0.181 | 0.183 | 0.177 | 0.180 | 0.187 | 0.173 | 0.168 | 0.186 | 0.166 | 0.192 | 0.199 | 0.199 | 0.248 |
| Spain | 0.180 | 0.183 | 0.180 | 0.168 | 0.173 | 0.174 | 0.136 | 0.147 | 0.167 | 0.164 | 0.183 | 0.246 | 0.268 |
| Sweden | 0.152 | 0.147 | 0.152 | 0.146 | 0.142 | 0.144 | 0.179 | 0.129 | 0.141 | 0.150 | 0.149 | 0.170 | 0.176 |
| Switzerland | 0.156 | 0.147 | 0.165 | 0.144 | **0.131** | 0.156 | NA | 0.158 | **0.127** | 0.155 | 0.160 | 0.176 | 0.169 |
| USA | 0.153 | 0.148 | 0.157 | 0.149 | 0.143 | 0.153 | 0.145 | 0.140 | 0.147 | 0.150 | 0.150 | 0.170 | 0.191 |

Note: Minimum and maximum values in each column are in bold letters. NAs are entered for subsamples including less than 50 observations.

Table S.3: Verbal fluency Gini estimates of the 50–84-year-olds, women 50-84 years old and men 50-84 years old, 60–74-year-olds, women 60-74 years old and men 60-74 years old, as well as by 5-year age groups between 50 and 84 for each country.

| **Country** | **50-84** | **50-84 Female** | **50-84 Male** | **60-74** | **60-74 Female** | **60-74 Male** | **50-54** | **55-59** | **60-64** | **65-69** | **70-74** | **75-79** | **80-84** |
| --- | --- | --- | --- | --- | --- | --- | --- | --- | --- | --- | --- | --- | --- |
| Austria | **0.045** | **0.042** | 0.049 | **0.032** | **0.032** | **0.034** | 0.051 | 0.043 | **0.023** | 0.035 | **0.047** | **0.073** | **0.087** |
| Belgium | 0.065 | 0.068 | 0.062 | 0.055 | 0.059 | 0.052 | 0.067 | 0.042 | 0.040 | 0.058 | 0.076 | 0.088 | 0.146 |
| Brazil | 0.262 | 0.259 | 0.263 | 0.261 | 0.260 | 0.262 | 0.247 | 0.259 | 0.264 | 0.260 | 0.258 | 0.276 | 0.301 |
| Bulgaria | 0.190 | 0.191 | 0.192 | 0.185 | 0.191 | 0.184 | NA | 0.201 | 0.183 | 0.192 | 0.198 | 0.197 | 0.238 |
| Chile | 0.159 | 0.158 | 0.162 | 0.145 | 0.145 | 0.149 | NA | NA | 0.133 | 0.154 | 0.159 | 0.190 | 0.238 |
| China | 0.214 | 0.217 | 0.210 | 0.219 | 0.220 | 0.217 | 0.204 | 0.207 | 0.211 | 0.223 | 0.229 | 0.234 | 0.243 |
| Croatia | 0.119 | 0.124 | 0.114 | 0.111 | 0.111 | 0.112 | 0.077 | 0.089 | 0.091 | 0.112 | 0.136 | 0.177 | 0.206 |
| Cyprus | 0.202 | 0.209 | 0.200 | 0.197 | 0.205 | 0.195 | NA | 0.193 | 0.188 | 0.190 | 0.233 | 0.231 | 0.205 |
| Czechia | 0.048 | 0.055 | **0.047** | 0.042 | 0.040 | 0.048 | 0.055 | 0.097 | 0.036 | 0.047 | 0.051 | 0.087 | 0.115 |
| Denmark | **0.045** | **0.042** | 0.050 | 0.038 | 0.034 | 0.043 | 0.035 | 0.039 | 0.037 | **0.030** | 0.050 | 0.074 | 0.102 |
| England | 0.077 | 0.076 | 0.078 | 0.071 | 0.067 | 0.075 | 0.063 | 0.046 | 0.071 | 0.061 | 0.081 | 0.112 | 0.150 |
| Estonia | 0.064 | 0.058 | 0.073 | 0.059 | 0.048 | 0.075 | 0.046 | 0.040 | 0.035 | 0.065 | 0.082 | 0.090 | 0.134 |
| Finland | 0.059 | 0.053 | 0.067 | 0.052 | 0.042 | 0.064 | NA | **0.033** | 0.049 | 0.051 | 0.065 | 0.085 | 0.155 |
| France | 0.065 | 0.067 | 0.065 | 0.061 | 0.059 | 0.064 | 0.051 | 0.054 | 0.051 | 0.061 | 0.077 | 0.098 | 0.116 |
| Germany | 0.059 | 0.058 | 0.061 | 0.055 | 0.051 | 0.060 | 0.036 | 0.038 | 0.046 | 0.054 | 0.070 | 0.093 | 0.121 |
| Ghana | 0.202 | 0.207 | 0.195 | 0.204 | 0.208 | 0.197 | 0.191 | 0.191 | 0.187 | 0.212 | 0.214 | 0.238 | 0.217 |
| Greece | 0.166 | 0.175 | 0.155 | 0.155 | 0.163 | 0.146 | 0.136 | 0.129 | 0.143 | 0.151 | 0.166 | 0.189 | 0.228 |
| Hungary | 0.136 | 0.133 | 0.141 | 0.136 | 0.132 | 0.144 | 0.122 | 0.123 | 0.121 | 0.138 | 0.154 | 0.164 | 0.177 |
| India | 0.197 | 0.201 | 0.188 | 0.198 | 0.201 | 0.189 | 0.188 | 0.189 | 0.193 | 0.197 | 0.206 | 0.218 | 0.224 |
| Indonesia | 0.189 | 0.198 | 0.175 | 0.201 | 0.214 | 0.178 | 0.173 | 0.176 | 0.182 | 0.206 | 0.238 | 0.237 | 0.255 |
| Ireland | 0.112 | 0.121 | 0.102 | 0.108 | 0.114 | 0.101 | 0.081 | 0.087 | 0.092 | 0.113 | 0.126 | 0.145 | 0.181 |
| Israel | 0.152 | 0.186 | 0.144 | 0.119 | 0.150 | 0.093 | NA | NA | NA | 0.143 | 0.133 | 0.174 | 0.262 |
| Italy | 0.172 | 0.175 | 0.172 | 0.155 | 0.160 | 0.152 | NA | 0.166 | 0.137 | 0.168 | 0.174 | 0.210 | 0.229 |
| Japan | NA | NA | NA | NA | NA | NA | NA | NA | NA | NA | NA | NA | NA |
| Latvia | 0.109 | 0.11 | 0.109 | 0.107 | 0.105 | 0.112 | 0.072 | 0.084 | 0.081 | 0.115 | 0.134 | 0.167 | 0.163 |
| Lithuania | 0.070 | 0.069 | 0.074 | 0.066 | 0.058 | 0.081 | NA | 0.044 | 0.061 | 0.06 | 0.091 | 0.112 | 0.167 |
| Luxembourg | 0.071 | 0.076 | 0.081 | 0.068 | 0.069 | 0.071 | NA | NA | 0.049 | 0.072 | 0.102 | 0.118 | NA |
| Malta | 0.145 | 0.153 | 0.142 | 0.130 | 0.136 | 0.129 | NA | 0.126 | 0.116 | 0.135 | 0.153 | 0.195 | 0.219 |
| Mexico | 0.153 | 0.154 | 0.152 | 0.157 | 0.158 | 0.156 | 0.129 | 0.136 | 0.143 | 0.161 | 0.170 | 0.184 | 0.209 |
| Netherlands | 0.063 | 0.069 | 0.072 | 0.049 | 0.053 | 0.055 | NA | 0.099 | 0.051 | 0.045 | 0.067 | 0.104 | 0.121 |
| Poland | 0.118 | 0.119 | 0.117 | 0.110 | 0.102 | 0.120 | 0.097 | 0.099 | 0.097 | 0.108 | 0.128 | 0.163 | 0.212 |
| Portugal | 0.183 | 0.200 | 0.174 | 0.165 | 0.179 | 0.183 | 0.272 | 0.161 | 0.188 | 0.167 | 0.207 | 0.221 | 0.246 |
| Romania | 0.128 | 0.137 | 0.121 | 0.129 | 0.139 | 0.121 | 0.119 | 0.099 | 0.123 | 0.121 | 0.159 | 0.169 | 0.221 |
| Russia | **0.325** | **0.303** | **0.361** | **0.319** | **0.303** | **0.352** | **0.279** | **0.321** | **0.310** | **0.312** | **0.350** | **0.382** | **0.345** |
| Slovakia | 0.165 | 0.170 | 0.161 | 0.180 | 0.179 | 0.185 | NA | 0.114 | 0.176 | 0.179 | 0.197 | 0.229 | 0.254 |
| Slovenia | 0.062 | 0.069 | 0.054 | 0.055 | 0.057 | 0.055 | **0.032** | 0.035 | 0.045 | 0.051 | 0.076 | 0.116 | 0.154 |
| South Africa | 0.212 | 0.209 | 0.217 | 0.225 | 0.216 | 0.238 | 0.196 | 0.206 | 0.230 | 0.218 | 0.238 | 0.199 | 0.305 |
| Spain | 0.167 | 0.179 | 0.155 | 0.150 | 0.157 | 0.153 | 0.171 | 0.159 | 0.150 | 0.149 | 0.159 | 0.215 | 0.231 |
| Sweden | 0.050 | 0.043 | 0.059 | 0.045 | 0.043 | 0.050 | 0.062 | **0.033** | 0.040 | 0.046 | 0.056 | 0.080 | 0.094 |
| Switzerland | 0.057 | 0.057 | 0.060 | 0.049 | 0.042 | 0.060 | NA | 0.047 | 0.042 | 0.054 | 0.066 | 0.094 | 0.110 |
| USA | 0.144 | 0.152 | 0.134 | 0.127 | 0.135 | 0.118 | NA | 0.130 | NA | 0.115 | 0.142 | 0.165 | 0.184 |

Note: Minimum and maximum values in each column are in bold letters. NAs are entered for subsamples including less than 50 observations.

Table S.4: Gini estimates compared to the estimated P80/P20 ratio for 50–84-year-olds.

|  | **Grip strength** | | **Immediate recall** | | **Verbal fluency** | |
| --- | --- | --- | --- | --- | --- | --- |
| **Country** | **Gini index** | **P80/P20** | **Gini index** | **P80/P20** | **Gini index** | **P80/P20** |
| Austria | 0.190 | 1.84 | 0.149 | 1.60 | 0.045 | 1.05 |
| Belgium | 0.208 | 1.88 | 0.159 | 1.40 | 0.065 | 1.18 |
| Brazil | 0.216 | 2.06 | 0.235 | 2.00 | 0.262 | 2.50 |
| Bulgaria | 0.210 | 1.90 | 0.188 | 1.75 | 0.190 | 2.00 |
| Chile | NA | NA | 0.222 | 2.00 | 0.159 | 1.82 |
| China | 0.229 | 2.00 | 0.182 | 1.75 | 0.214 | 2.12 |
| Croatia | 0.202 | 1.96 | 0.202 | 1.75 | 0.119 | 1.43 |
| Cyprus | 0.197 | 1.90 | 0.180 | 1.50 | 0.202 | 2.22 |
| Czechia | 0.195 | 1.80 | 0.149 | 1.40 | 0.048 | 1.05 |
| Denmark | 0.188 | 1.85 | 0.154 | 1.40 | 0.045 | 1.05 |
| England | 0.199 | 1.91 | 0.148 | 1.60 | 0.077 | 1.18 |
| Estonia | 0.198 | 1.88 | 0.163 | 1.40 | 0.064 | 1.11 |
| Finland | 0.179 | 1.84 | 0.154 | 1.40 | 0.059 | 1.11 |
| France | 0.196 | 1.92 | 0.153 | 1.75 | 0.065 | 1.18 |
| Germany | 0.182 | 1.84 | 0.151 | 1.40 | 0.059 | 1.11 |
| Ghana | 0.236 | 1.95 | 0.175 | 1.50 | 0.202 | 2.00 |
| Greece | 0.193 | 1.75 | 0.174 | 1.75 | 0.166 | 1.82 |
| Hungary | 0.200 | 1.82 | 0.153 | 1.40 | 0.136 | 1.67 |
| India | 0.196 | 1.91 | 0.201 | 1.50 | 0.197 | 1.88 |
| Indonesia | 0.194 | 1.83 | 0.268 | 2.50 | 0.189 | 1.80 |
| Ireland | 0.205 | 1.90 | 0.169 | 1.75 | 0.112 | 1.43 |
| Israel | 0.234 | 2.00 | 0.205 | 1.75 | 0.152 | 1.54 |
| Italy | 0.184 | 1.75 | 0.171 | 1.50 | 0.172 | 1.82 |
| Japan | 0.172 | 1.80 | 0.236 | 2.00 | NA | NA |
| Latvia | 0.186 | 1.80 | 0.153 | 1.75 | 0.109 | 1.43 |
| Lithuania | 0.188 | 1.76 | 0.182 | 1.50 | 0.07 | 1.18 |
| Luxembourg | 0.190 | 1.76 | 0.170 | 1.60 | 0.071 | 1.18 |
| Malta | 0.198 | 1.86 | 0.181 | 1.50 | 0.145 | 1.67 |
| Mexico | 0.185 | 1.70 | NA | NA | 0.153 | 1.67 |
| Netherlands | 0.184 | 1.77 | 0.155 | 1.75 | 0.063 | 1.11 |
| Poland | 0.182 | 1.83 | 0.188 | 1.50 | 0.118 | 1.43 |
| Portugal | 0.188 | 1.77 | 0.234 | 2.00 | 0.183 | 2.00 |
| Romania | 0.182 | 1.83 | 0.181 | 1.50 | 0.128 | 1.54 |
| Russia | 0.230 | 1.96 | 0.180 | 1.75 | 0.325 | 3.00 |
| Slovakia | 0.201 | 1.81 | 0.214 | 1.75 | 0.165 | 2.00 |
| Slovenia | 0.190 | 1.85 | 0.162 | 1.75 | 0.062 | 1.18 |
| South Africa | 0.281 | 2.27 | 0.181 | 1.75 | 0.212 | 1.86 |
| Spain | 0.199 | 1.95 | 0.180 | 1.50 | 0.167 | 1.82 |
| Sweden | 0.187 | 1.85 | 0.152 | 1.40 | 0.050 | 1.05 |
| Switzerland | 0.180 | 1.88 | 0.156 | 1.40 | 0.057 | 1.11 |
| USA | 0.187 | 1.85 | 0.153 | 1.40 | 0.144 | 1.67 |


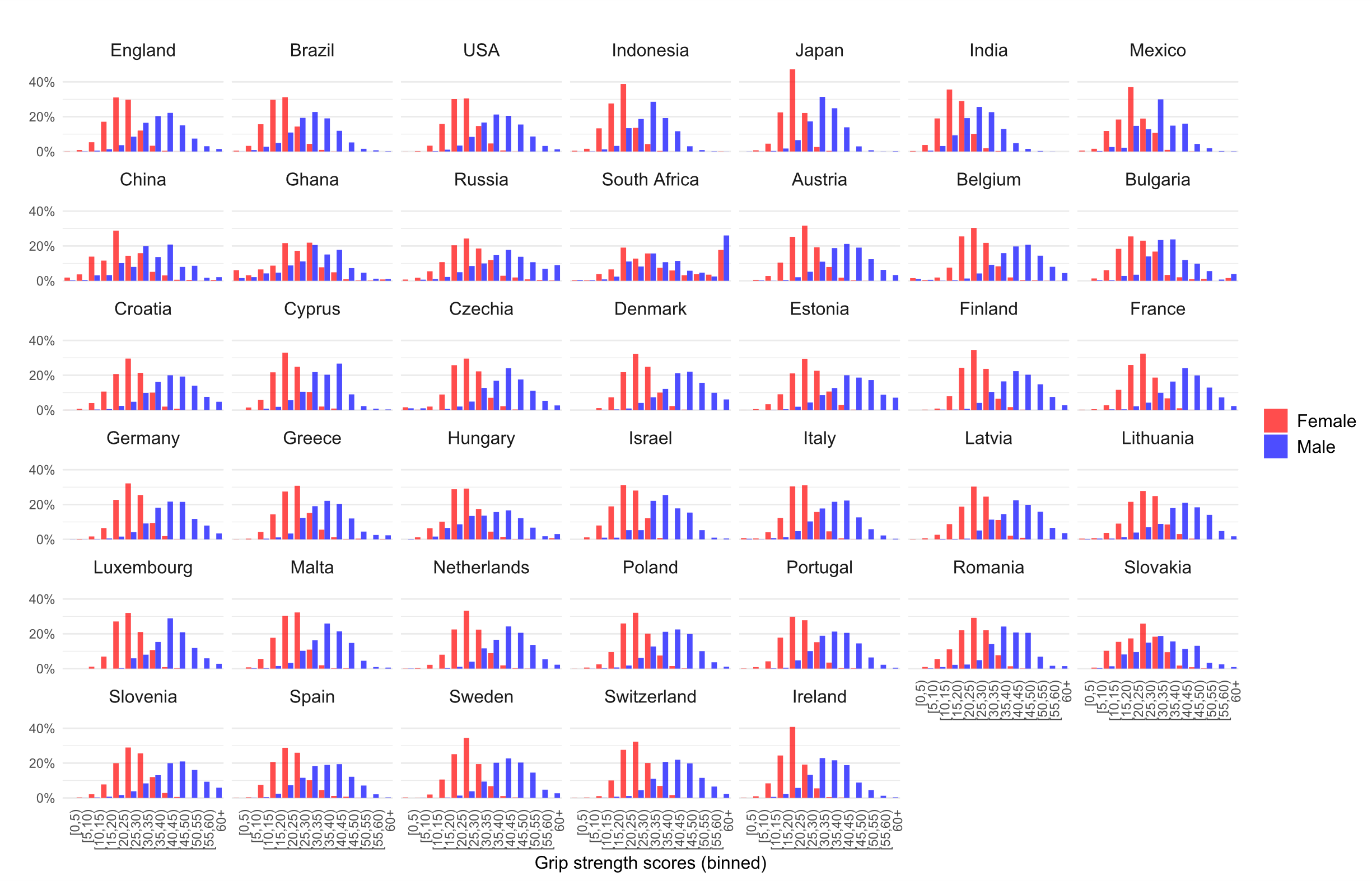


Figure S.1: Grip strength test score distribution as percentage of the sample of older adults aged 50 to 84 per country by gender.

Note: Countries are ordered alphabetically by survey name.


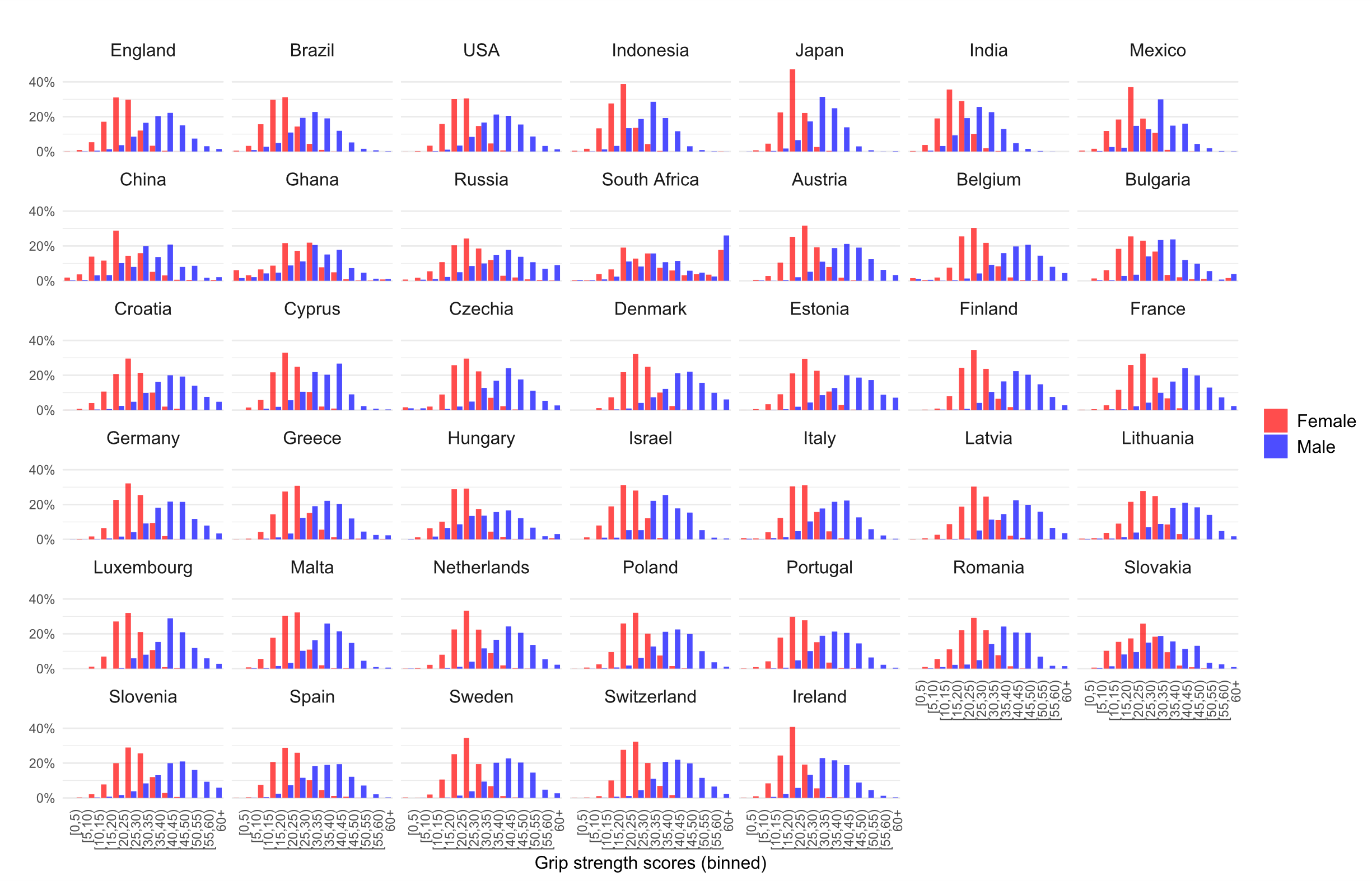

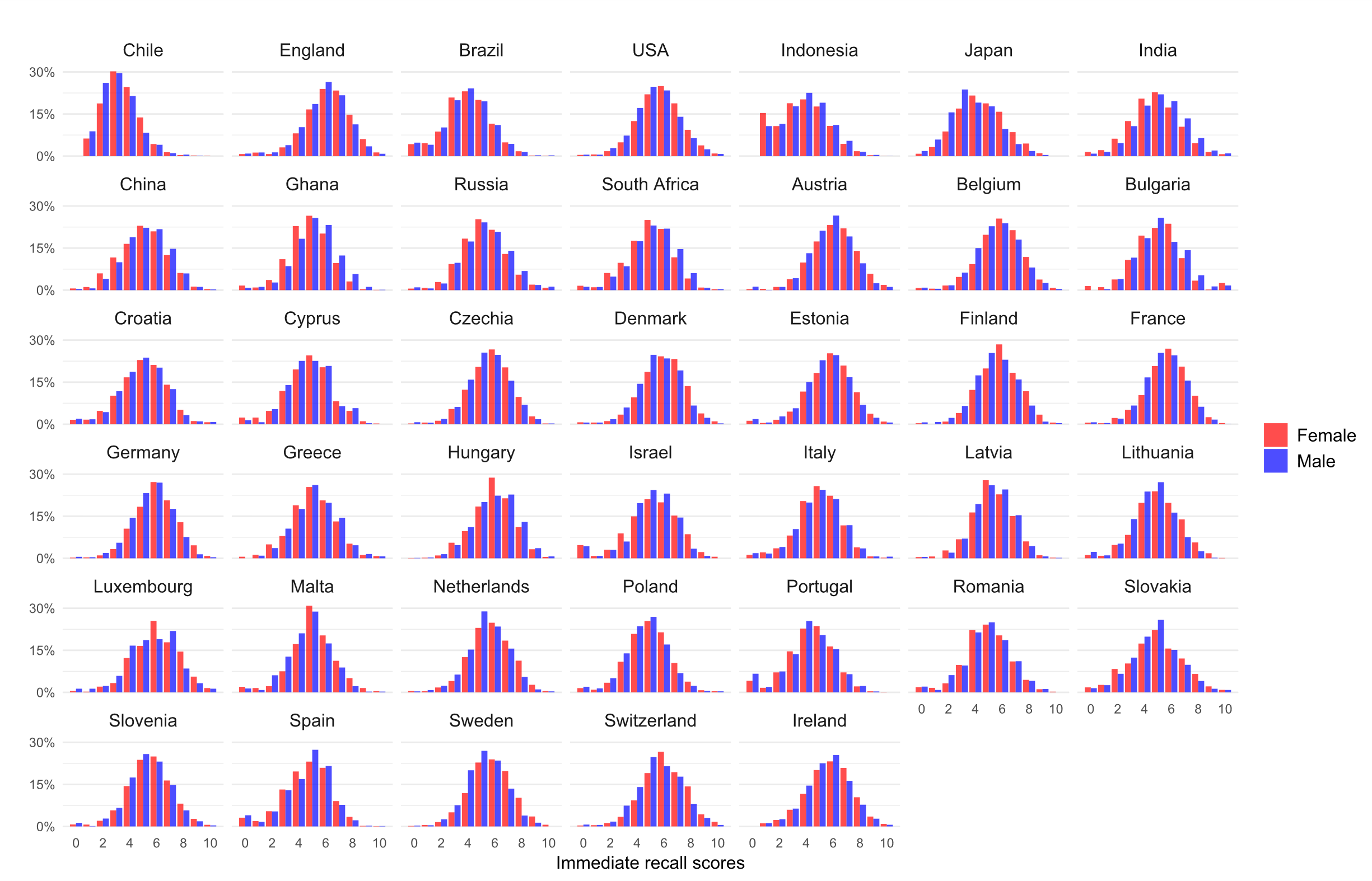


Figure S.1: Grip strength test score distribution as percentage of the sample of older adults aged 50 to 84 per country by gender.

Figure S.2: Immediate recall test score distribution as percentage of the sample of older adults aged 50 to 84 per country by gender.

Note: Countries are ordered alphabetically by survey name.


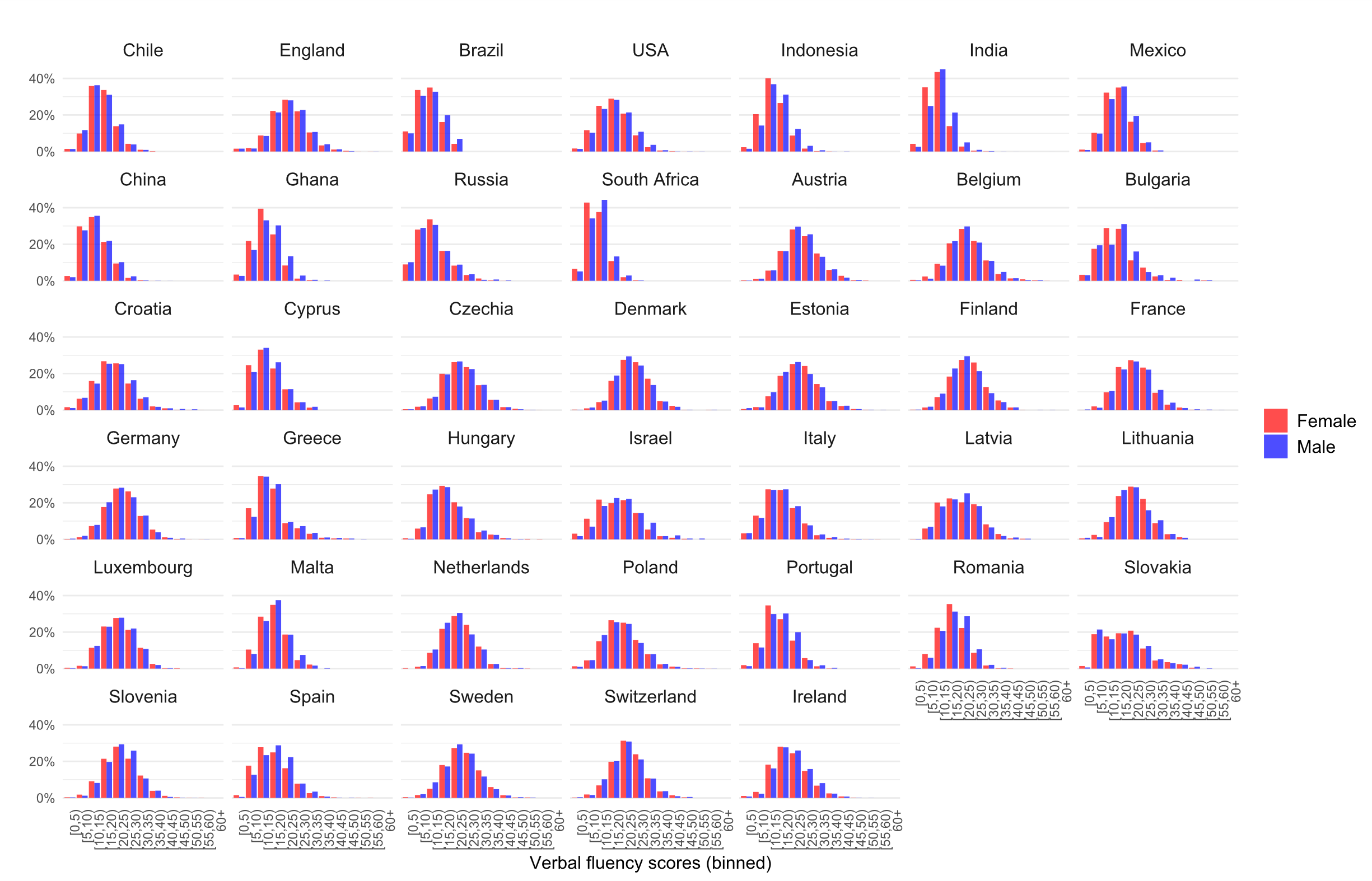


Note: Countries are ordered alphabetically by survey name.

Figure S.3: Verbal fluency test score distribution as percentage of the sample of older adults aged 50 to 84 per country by gender.

Figure S.4: Gini estimates for handgrip strength, immediate recall and verbal fluency by age group and country.


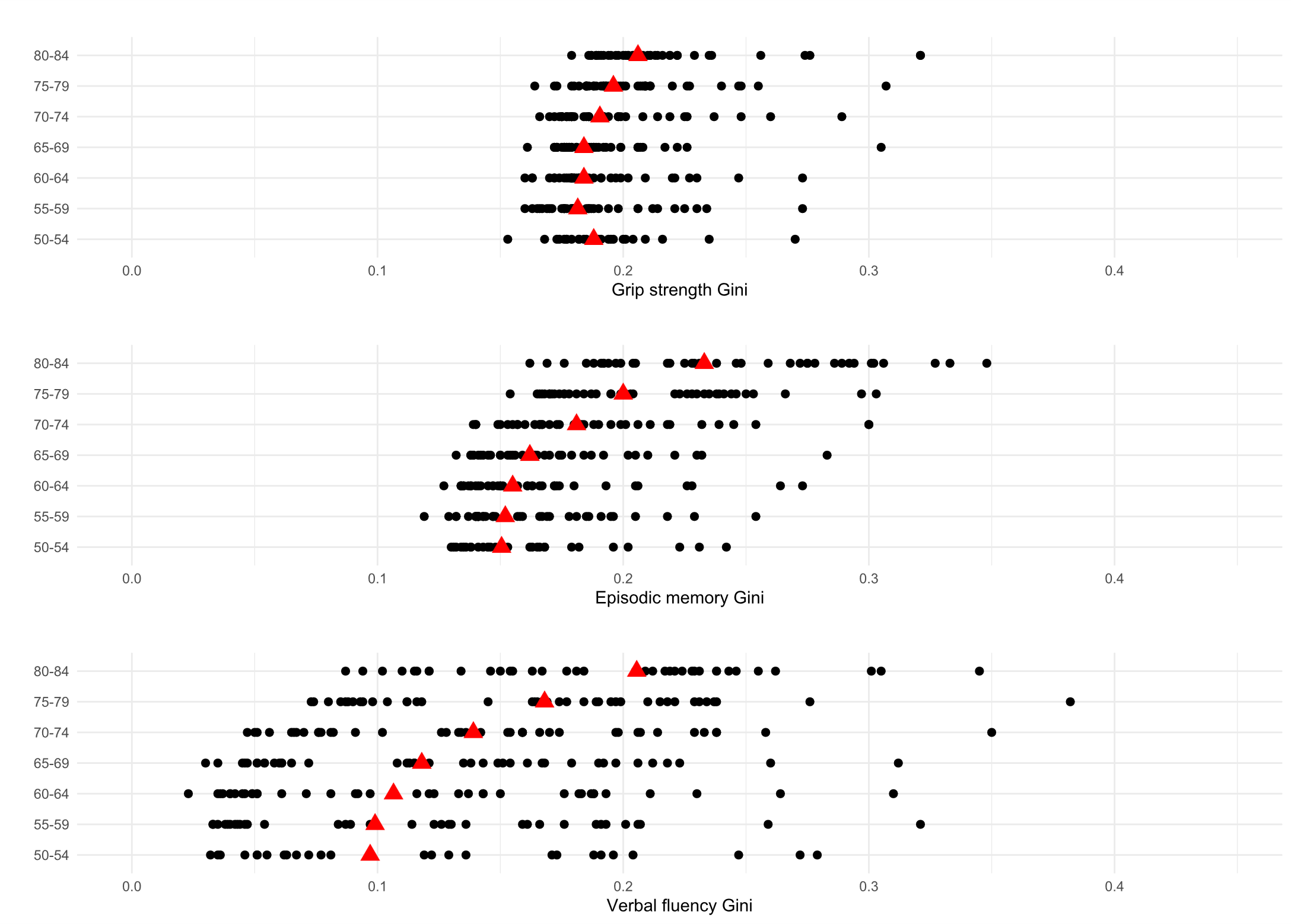


Note: Each black dot represents a country, the red triangles denote the median Gini estimates across the countries in the respective age group.


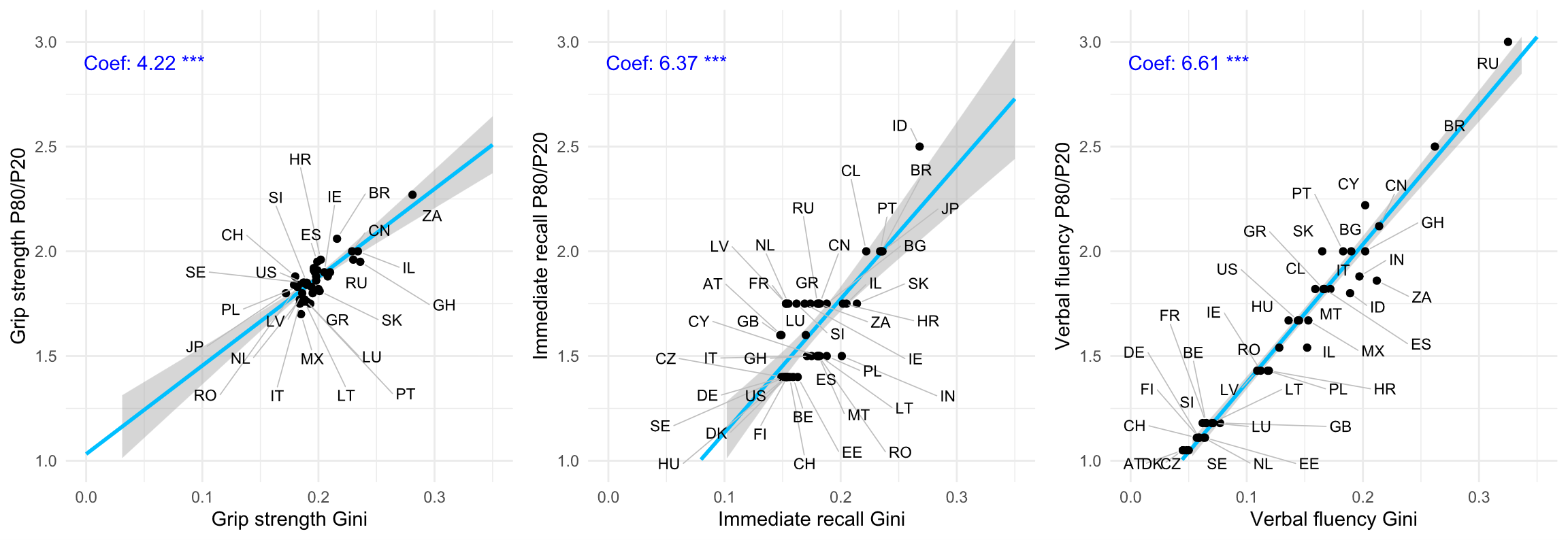


Figure S.5: The relationship between the Gini index and the P80/P20 ratio on the country level for the sample aged 50 to 84.

Note: The upper left of the tables shows the coefficients and their significance levels when regressing the P80/P20 ratio on the Gini (*** p<0.001). The blue line represents a regression line, with the grey area denoting the standard errors.


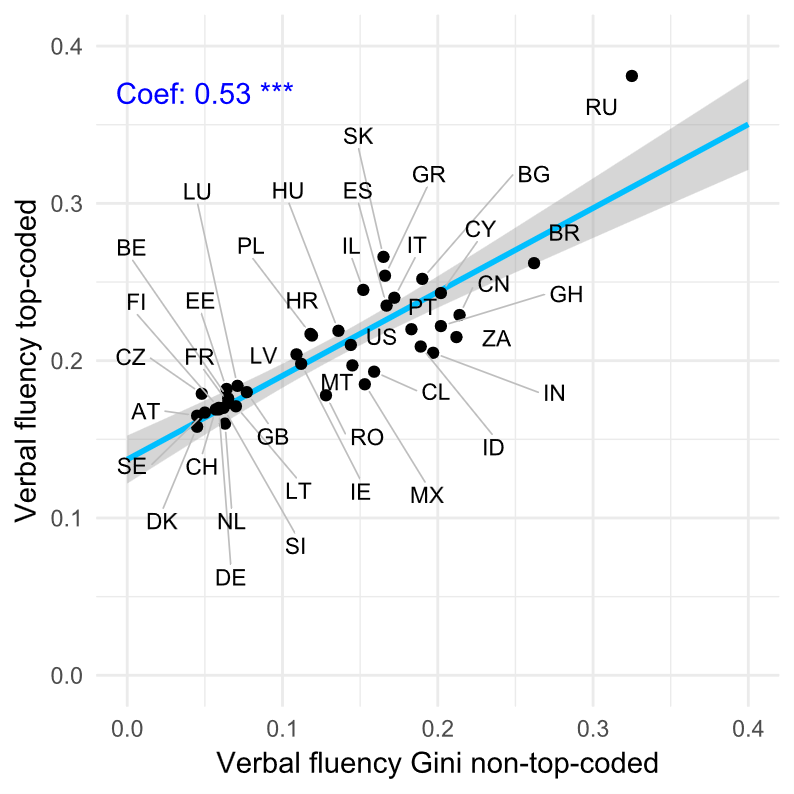


Note: The upper left of the tables shows the coefficients and their significance levels when regressing the top-coded Gini on the Gini (*** p<0.001). The blue line represents a regression line, with the grey area denoting the standard errors.

Figure S.6: The relationship between the Verbal fluency Gini index and the non-top-coded verbal fluency Gini index on the country level for the sample aged 50 to 84.
